# Identifying Heart Failure in ECG Data With Artificial Intelligence—A Meta-Analysis

**DOI:** 10.3389/fdgth.2020.584555

**Published:** 2021-02-25

**Authors:** Dimitri Grün, Felix Rudolph, Nils Gumpfer, Jennifer Hannig, Laura K. Elsner, Beatrice von Jeinsen, Christian W. Hamm, Andreas Rieth, Michael Guckert, Till Keller

**Affiliations:** ^1^Department of Internal Medicine I, Cardiology, Justus-Liebig University Giessen, Giessen, Germany; ^2^Cognitive Information Systems, KITE - Kompetenzzentrum für Informationstechnologie, Technische Hochschule Mittelhessen - University of Applied Sciences, Friedberg, Germany; ^3^Department of Cardiology, Kerckhoff Heart and Thorax Center, Bad Nauheim, Germany; ^4^Department of MND - Mathematik, Naturwissenschaften und Datenverarbeitung, Technische Hochschule Mittelhessen - University of Applied Sciences, Friedberg, Germany

**Keywords:** artificial intelligence, heart failure, diagnosis, ECG, meta-analysis

## Abstract

**Introduction:** Electrocardiography (ECG) is a quick and easily accessible method for diagnosis and screening of cardiovascular diseases including heart failure (HF). Artificial intelligence (AI) can be used for semi-automated ECG analysis. The aim of this evaluation was to provide an overview of AI use in HF detection from ECG signals and to perform a meta-analysis of available studies.

**Methods and Results:** An independent comprehensive search of the PubMed and Google Scholar database was conducted for articles dealing with the ability of AI to predict HF based on ECG signals. Only original articles published in peer-reviewed journals were considered. A total of five reports including 57,027 patients and 579,134 ECG datasets were identified including two sets of patient-level data and three with ECG-based datasets. The AI-processed ECG data yielded areas under the receiver operator characteristics curves between 0.92 and 0.99 to identify HF with higher values in ECG-based datasets. Applying a random-effects model, an sROC of 0.987 was calculated. Using the contingency tables led to diagnostic odds ratios ranging from 3.44 [95% confidence interval (CI) = 3.12–3.76] to 13.61 (95% CI = 13.14–14.08) also with lower values in patient-level datasets. The meta-analysis diagnostic odds ratio was 7.59 (95% CI = 5.85–9.34).

**Conclusions:** The present meta-analysis confirms the ability of AI to predict HF from standard 12-lead ECG signals underlining the potential of such an approach. The observed overestimation of the diagnostic ability in artificial ECG databases compared to patient-level data stipulate the need for robust prospective studies.

## Introduction

Heart failure (HF) is a common, yet unfavorable, cardiac condition. Up to 20% of all individuals in developed countries develop HF within their lifetime, and a large proportion of patients hospitalized for HF dies within 1 year of diagnosis (1).

Evaluation of symptoms suggestive of HF currently demands physicians to valuate various parameters including imaging and laboratory data and the electrocardiogram (ECG). Besides a standard examination that includes an ECG, imaging information, such as echocardiography or magnetic resonance imaging, is seen as gold standard in diagnosis of HF (2). Nevertheless, an adequate use of such imaging data is associated with relevant technical infrastructure and medical expertise. The ECG is a well-established, quick, and easily accessible method for diagnosis and screening of various cardiovascular diseases. It provides specific features that indicate presence of HF or prognosis in HF patients especially to rule out HF in case of a normal ECG (3, 4). However, use of an ECG as primary diagnostic instrument often only yields insufficient diagnostic specificity (5). Further, general practitioner–based ECG reporting has varying results, introducing further diagnostic uncertainty (6).

Devices providing medically relevant information generated directly by individuals outside the healthcare system such as smartphones with health applications or wearables including smartwatches are an emerging trend. This development promises that a growing number of, e.g., ECG data generated at home will be available for a diagnostic screening. Such data have already shown potential in computer-aided decision support systems to warn patients of rhythmic abnormalities (7). Management of this quantity of data, however, might be a challenge for the individual healthcare professional, as well as for the healthcare system itself. The potentially beneficial use of artificial intelligence (AI) in cardiology in general has been discussed already, e.g., as a tool for clinicians that could facilitate precision in daily practice and even might improve patient outcomes (8). AI might also be able to help in interpretation of ECG signals and could therefore be used to analyze ECG data in specific cases and on a large scale for early identification of cardiovascular diseases such as HF (9). Few studies have performed analyses of AI systems to detect HF from ECG data. In these studies, the methods and patient numbers vary strongly. The aim of the present evaluation was to perform a meta-analysis on these studies and thereby give an overview on the current possibilities of the use of AI in automated HF detection from ECG signals.

## Methods

**Figure 1:**
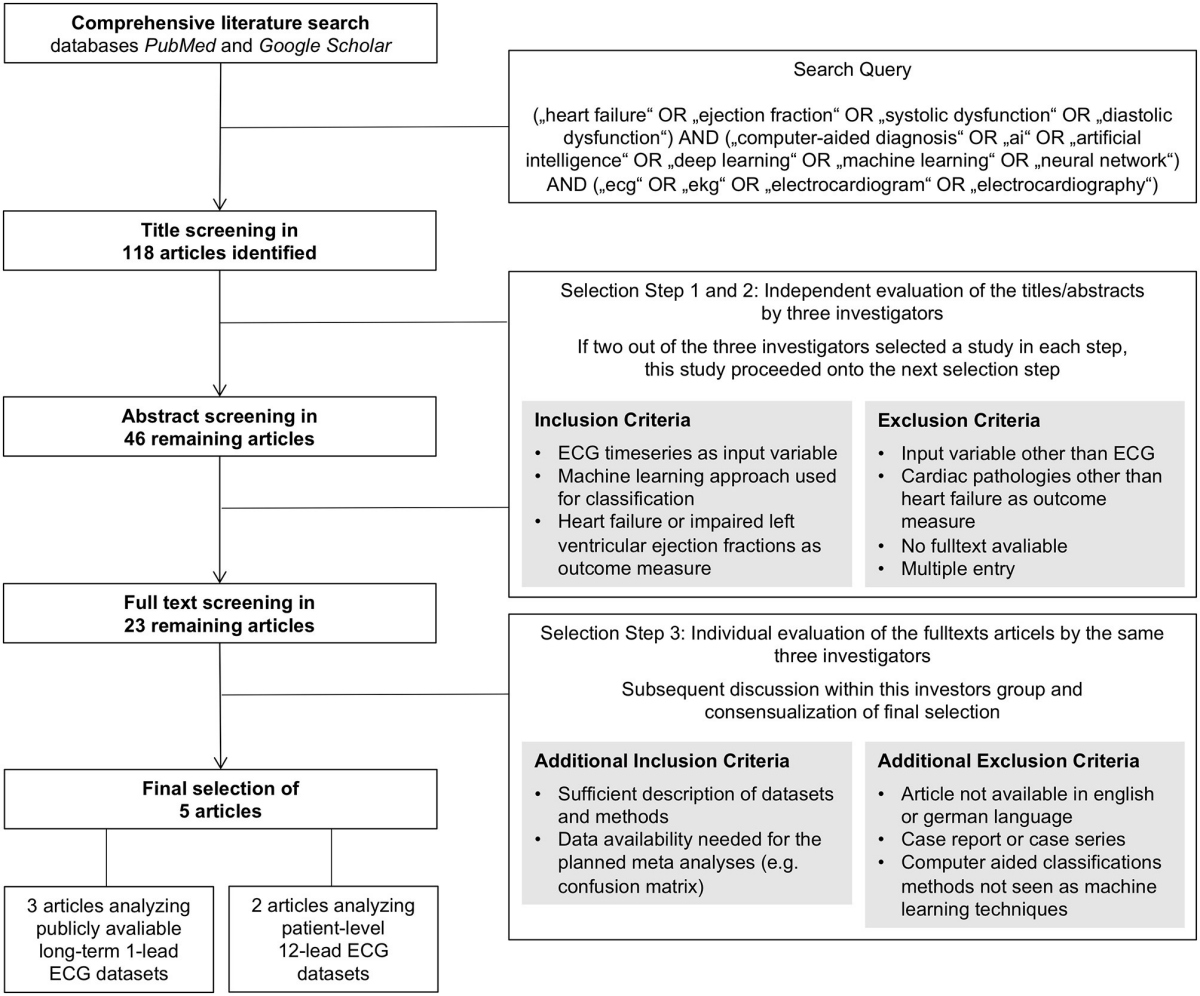
Flowchart summarizing the literature screening and study selection process.

A comprehensive literature search for original articles on the ability of AI to predict HF based on ECG signals was conducted using the databases PubMed and Google Scholar on May 13, 2020. These two databases were searched using the following keyword combinations as search query: (“heart failure” OR “ejection fraction” OR “systolic dysfunction” OR “diastolic dysfunction”) AND (“computer-aided diagnosis” OR “ai” OR “artificial intelligence” OR “deep learning” OR “machine learning” OR “neural network”) AND (“ecg” OR “ekg” OR “electrocardiogram” OR “electrocardiography”). The term “computer-aided” was added to the query to not miss articles that use a more general title potentially not revealing an AI approach as basis for a computer-based classification algorithm. This search query led to a list of 118 titles that were further screened and selected by three of the authors (D.G., F.R., and T.K.). As primary endpoints, the criteria congestive HF and reduced left ventricular ejection fraction [left ventricular ejection fraction (LVEF) ≤40%] were used. Identification of this endpoint had to be based on ECG time-series data as input by an AI approach. Artificial neural networks, support vector machines, random forest classifiers, and k-nearest neighbor algorithms qualified as an AI approach in this context. The screening and selection process was carried out in three steps: first a title, then an abstract, and finally a full text screening and selection. Evaluation of studies within the first and second steps was conducted by the three mentioned investigators independently. A study was selected for evaluation within the next step if at least two of the three investigators selected the individual study. After abstract classification, a total of 23 studies were selected for full text assessment. The subsequent third step was conducted by the same three investigators independently, followed by a discussion within the investigator team and a consensual selection of the articles to be evaluated within the meta-analysis. Within this third step, the quality of the studies was assessed oriented on the PRISMA (Preferred Reporting Items for Systematic Reviews and Meta-Analyses) statement (10). Further, data availability of the needed information, e.g., reporting of a confusion matrix, was checked. The final set of studies consisted of five articles that fulfilled the defined criteria and provided sufficient information for the subsequent data extraction enabling the meta-analysis. This selection process including the applied criteria is also depicted with a flowchart as Figure 1.

To assess the heterogeneity between the selected studies, the DerSimonian-Laird estimator (τ^2^) and *I*^2^ statistics were used (11, 12). Within the meta-analysis, principal measurement of effect size was the diagnostic odds ratio (DOR) after natural logarithmic transformation (lnDOR) with 95% confidence interval (CI). For univariate analyses, a random-effects model was used. For the bivariate analyses, a summary receiver operating characteristics (sROC) curve was constructed, and a summary area under the ROC curve was calculated. For descriptive reasons, for the studies that did not provide these data, an AUC was estimated based on the respective contingency table (13–15). All statistical analyses were carried out using R3.6.0 with the *meta* (V4.12-0) and the *mada* (V0.5.10) packages (R Foundation for Statistical Computing, Vienna, Austria).

## Results

The five evaluated studies comprise a total of 57,027 patients and 579,134 ECG datasets. Two of these studies, both published by Attia et al. are based on patient-level data with large cohort sizes of 3,874 and of 52,870 individuals, reflecting a clinical application of an AI-based diagnostic approach (16, 17). These cohorts comprised unselected patients who underwent routine ECG and available echocardiographic data with the endpoint LVEF ≤35%. The other three studies used large numbers of ECG datasets as basis stemming from only a small number of individuals (33–107). These ECG datasets were taken from different existing databases such as the publicly available Fantasia or BIDMC database used in all three evaluated publications (18–20). Here, endpoint was the classification as congestive HF provided within these databases.

Four studies used the raw ECG time-series data as input with 500 to 12 × 1,000 features comprising the input of the respective algorithms (14–17), whereas one study used five extracted features as input (13). The proposed respective computer-aided diagnostic algorithms used a convolutional neural network (CNN) in three publications (14, 16, 17), a CNN plus long short-term memory network in one publication (15), and a dual-tree complex wavelet transform (DTCWT) model in one publication (13). The latter was accepted as an AI approach for this meta-analysis as all other criteria were fulfilled even if DTCWT itself would not qualify according to the predefined AI methods.

The algorithms of the five evaluated studies were associated with sensitivities ranging from 83 to 100% and specificities ranging from 86 to 100% identifying HF with higher values in ECG dataset–based studies. Table 1 provides an overview of the five evaluated studies.

**Table 1:** Summary of the studies included in the meta-analysis.

**Study**	**Classification method**	**Input features**	**Outcome measure**	**No. of patients**	**No. of ECGs**	**Classification performance**
Sudarshan et al. (13)	DTCWT	Five features based on 2-s segments of one-lead long-term ECG recordings	CHF	Set1: 55 Set2: 33	Set1: 82,427 Set2: 84,952	Sens (1): 1.00 (95% CI = 1.00–1.00) Spec (1): 1.00 (95% CI = 1.00–1.00) Sens (2): 0.97 (95% CI = 0.97–0.97) Spec (2): 0.99 (95% CI = 0.99–0.99)
Acharya et al. (14)	CNN	500 features based on 2-s segments of one-lead long-term ECG recordings	CHF	Set1: 33 Set2: 55	Set1: 100,308 Set2: 140,000	Sens (1): 0.97 (95% CI0.96–0.97) Spec (1): 0.96 (95% CI = 0.96–0.96) Sens (2): 0.99 (95% CI = 0.99–0.99) Spec (2): 0.99 (95% CI = 0.99–0.99)
Attia et al. (17)	CNN	12 × 1,000 features (zero-padded to 1,024) based on a 2-s segment from 10-s 12-lead ECG recordings	Low LVEF	52,870	52,870	Sens: 0.83 (95% CI = 0.78–0.87) Spec: 0.87 (95% CI = 0.86–0.88)
Attia et al. (16)	CNN	12 × 1,000 features (zero-padded to 1,024) based on a 2-s segment from 10-s 12-lead ECG recordings	Low LVEF	3,874	3,874	Sens: 0.86 (95% CI = 0.85–0.87) Spec: 0.86 (95% CI = 0.85–0.86)
Lih et al. (15)	CNN-LSTM	2,000 features based on 2-s segments of one-lead long-term ECG recordings	CHF (+ MI, CAD)	107	114,703	Sens: 0.99 (95% CI = 0.99–0.99) Spec: 0.98 (95% CI = 0.98–0.98)

*DTCWT, dual-tree complex wavelet transform; CNN, convolutional neural network; LSTM, long short-term memory; CHF, congestive heart failure; LVEF, left ventricular ejection fraction; MI, myocardial infarction; CAD, coronary artery disease; Sens, sensitivity; Spec, specificity*.

**Figure 2:**
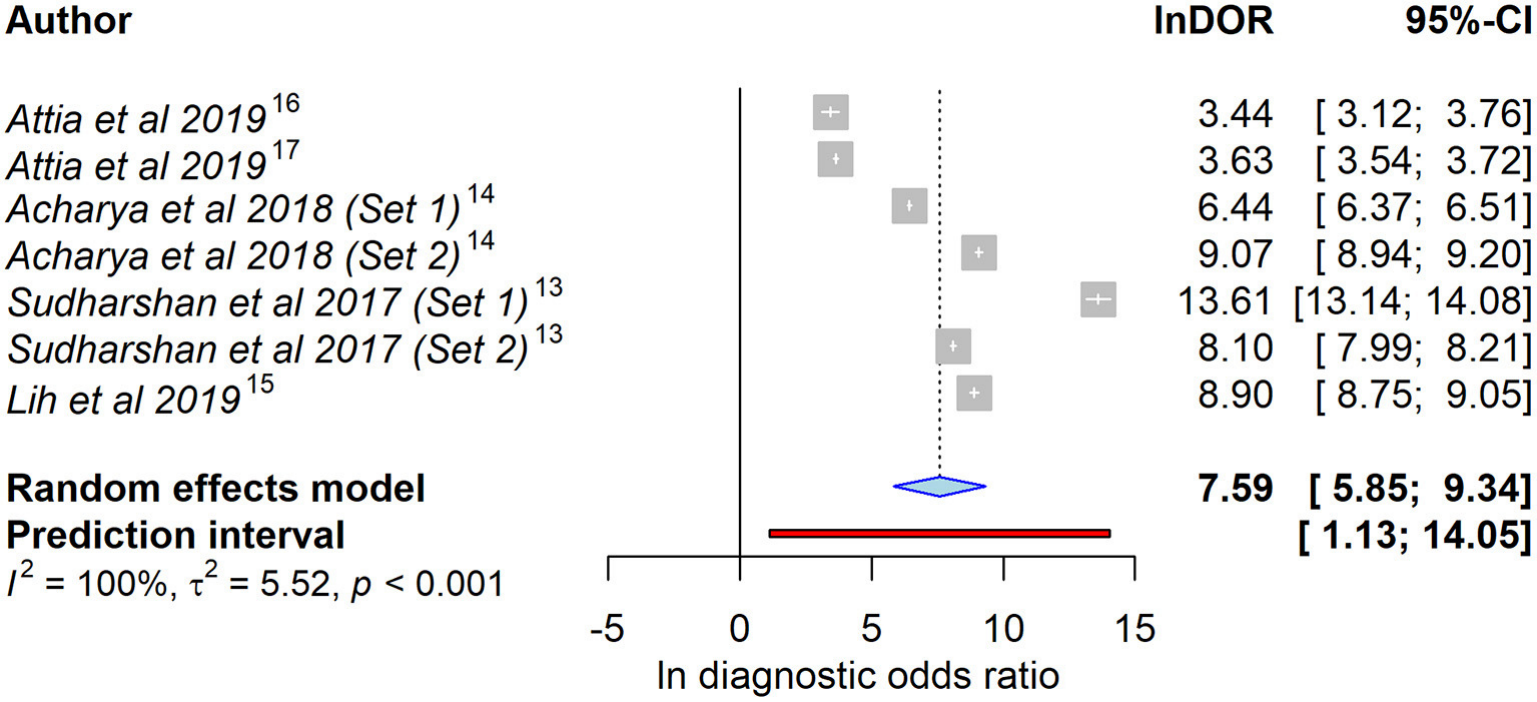
Forest plot of the selected studies showing the ability to identify heart failure using artificial intelligence–processed ECG data. Data presented as a univariate analysis using a random-effects model with diagnostic odds ratio after natural logarithmic transformation (lnDOR) with respective confidence interval (CI).

**Figure 3:**
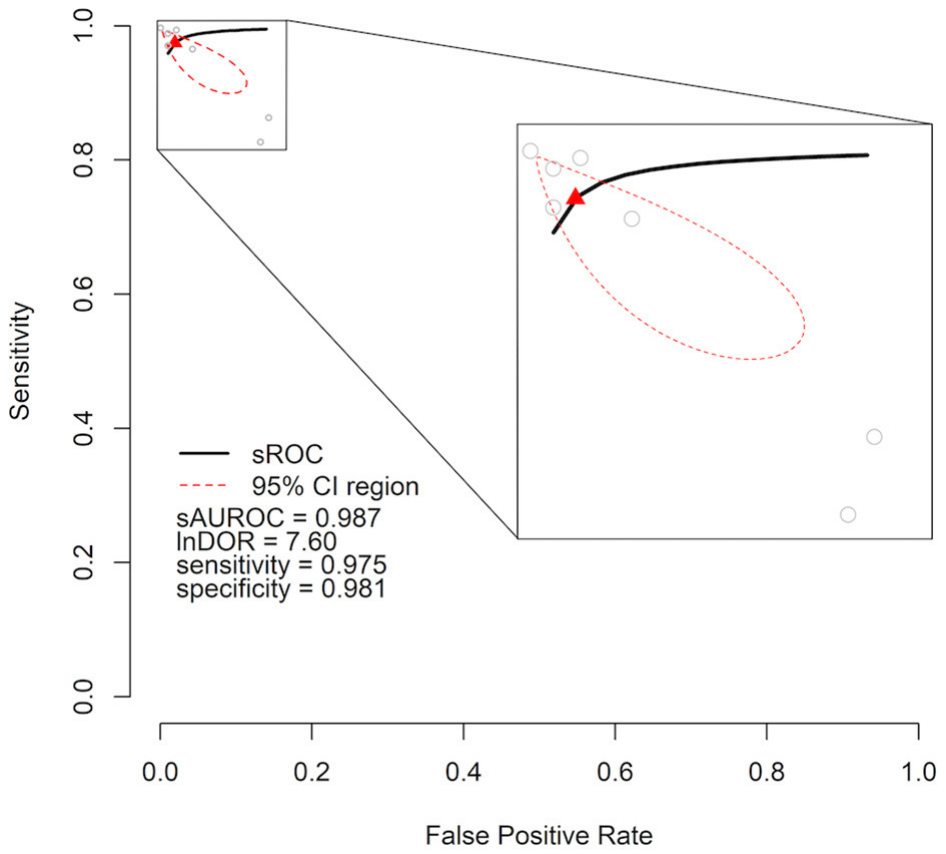
Cumulative summary receiver operating characteristic curve (sROC) of an artificial intelligence–processed ECG approach to detect heart failure. Individual studies are shown as gray circles. Summary point is shown as red triangle. The area of interest is magnified on the right side. lnDOR denotes diagnostic odds ratio after natural logarithmic transformation, sAUROC denotes area under the sROC curve; CI, confidence interval.

As meta-analysis, we calculated a combined DOR of 7.59 (95% CI = 5.85–9.34) after log transformation. This high lnDOR reflects the lnDORs of the individual studies starting from 3.44 (95% CI = 3.12–3.76) up to 13.61 (95% CI = 13.14–14.08) with lower diagnostic performance in patient-level datasets (Figure 2). For the bivariate analysis, an sROC curve was calculated, leading to a combined area under the curve of 0.987. Again, the diagnostic performance was lower in patient-level studies with an area under the curve of 0.92 and 0.93 compared to 0.96, 0.99, 0.99, 0.98, and 0.99 (Figure 3). This observed heterogeneity between the individual studies is reflected by a τ^2^ of 5.52 and *I*^2^ of 100% (*p* < 0.001).

## Discussion and Conclusions

The observed diagnostic information of an AI approach using ECG data to identify HF in our meta-analysis confirms the potential of computer-aided decision-making using ECG data in diagnoses other than arrhythmias. Our analysis further shows a relevant heterogeneity between studies based on ECG data and studies based on patient-level datasets suggesting that a meta-analysis incorporating both study types might not be as meaningful as desired. Further limitation for a meta-analysis of these five studies is the varying endpoint. Still, the individual results of the studies itself all show promising results pointing in the same direction supporting the information of the meta-analysis.

Three publications of our meta-analysis are based on cases from one-lead long-term ECG recordings of the BIDMC congestive HF database, which consists of only 15 patients (13–15). Those recordings were segmented into short 2-s intervals to artificially increase the number of datasets.

In contrast, the studies of Attia et al. used 2-s segments stemming from standard 12-lead ECGs with a length of 10 s obtained in 3,874 and 52,870 individual patients, respectively (16, 17). These datasets might better depict real-life data as analyses of the segmented ECGs seem to overestimate the ability of AI to detect HF in comparison. These patient-based datasets still show a clinically relevant diagnostic information with an AUC of > 0.8. This assumption is further supported by a study by Kwon et al. who reported comparable patient-based dataset AUCs of 0.843 and 0.889 for two datasets (3,378 and 5,901 patients) (21). Interestingly, the used datasets, here patient-based vs. ECG-based, had a larger impact on the model performance compared to a difference in input features. Using ECG datasets, the study by Sudarshan et al. (13) with only five features, yielded a comparable classification performance to the studies by Acharya et al. (14) with 500 input features, and Lih et al. (15) with 2,000 input features.

ECG characteristics are known to vary according to ethnicity, possibly impacting the accuracy of an AI algorithm that was trained with datasets stemming from specific geographical regions. Using the same dataset as Attia et al. (16, 17), Noseworthy et al. found that, while varying accuracies between ethnic groups are present, their network performed consistently across multiple ethnicities (22).

Besides ECG data, other information available after a recommended clinical diagnostic workup (2) might also be a valid input for an AI approach. Here, the use of data stemming from classical imaging techniques such as chest X-rays (23) or from the gold-standard imaging method of echocardiography (24) has shown a relevant potential. Also, traditional diagnostic methods, not relying on a complex infrastructure, like the evaluation of heart sound via a computer-aided approach (25), might be of use in the evaluation of HF patients. Further, combination of such different modalities as input features compared to a single diagnostic method might increase model precision in a real-world setting. Such an idea is supported by data showing that various information taken from electronic health records within a machine learning approach is able to predict HF before it is clinically obvious (26). With the inhomogeneous nature regarding features as well as outcome measures in AI-aided HF diagnosis, this analysis focuses on ECG time series as input variable. Nevertheless, other input parameters and the combination of different modalities have to be addressed by future studies.

The present meta-analysis, as well as the published data, underlines the need for robust large patient-level data–based studies to better appraise the value of AI in ECG interpretation in the context of HF. Here, the ongoing ECG AI-Guided Screening for Low Ejection Fraction (EAGLE) cluster randomized trial (NCT04000087) will provide useful prospective insights representing a real-life setting (27, 28).

Recently, technology and acceptance of wearables, smart-health devices, and applications have widely improved. The growing processing power and system memory will diminish technical limitations. Especially, one-lead ECG assessment has been implemented as feature into several devices. Supporting our observations regarding different types of ECG input, promising data on the transferability of a neural network trained with 12-lead ECGs to a one-lead ECG–enabled device have been presented at the annual meeting of the American Heart Association in 2019 underlining the potential of such an approach (29).

To conclude, the data of this meta-analysis confirm a substantial ability of AI to predict HF or a reduced LVEF from standard ECG signals. With the current advances of mobile devices capable of ECG recording, AI might be a powerful future tool in screening for HF or even diagnosis of other diseases of the heart.

## Data Availability Statement

The original contributions presented in the study are included in the article/supplementary material, further inquiries can be directed to the corresponding author/s.

## Ethics Statement

Ethical review and approval was not required for the study on human participants in accordance with the local legislation and institutional requirements. Written informed consent for participation was not required for this study in accordance with the national legislation and the institutional requirements.

## Author Contributions

Conception and design of the work was done by DG, FR, and TK. Data was collected by DG, FR, and TK. Data analyses were done by DG, NG, and JH. DG and FR visualized the data. The draft of the manuscript was created by DG, FR, and TK. MG and TK supervised the project. NG, JH, LE, BJ, CH, AR, and MG contributed to the interpretation of the result and critically revised the manuscript. All authors gave approval of the final version of the manuscript.

## Conflict of Interest

The authors declare that the research was conducted in the absence of any commercial or financial relationships that could be construed as a potential conflict of interest.
